# Outcome of in vitro fertilization in women with subclinical hypothyroidism

**DOI:** 10.1186/s12958-017-0257-2

**Published:** 2017-05-25

**Authors:** YunYing Cai, LanPing Zhong, Jie Guan, RuiJin Guo, Ben Niu, YanPing Ma, Heng Su

**Affiliations:** 1 Faculty of Environmental Science and Engineering, Kunming University of Science and Technology, Kunming, Yunnan Province 650500 People’s Republic of China; 2 Department of Endocrinology, The Affiliated Hospital of Kunming University of Science and Technology, The First People’s Hospital of Yunnan, Kunming, Yunnan Province 650032 People’s Republic of China; 3 Reproductive Medicine Center, The Affiliated Hospital of Kunming University of Science and Technology, The First People’s Hospital of Yunnan, Kunming, Yunnan 650032 People’s Republic of China

**Keywords:** Thyroid function, in vitro fertilization, Controlled ovarian stimulation, Subclinical hypothyroidism, Infertility

## Abstract

**Background:**

Previous studies examining associations between subclinical hypothyroidism (SCH) with in vitro fertilization (IVF) outcome indicate some benefits of levothyroxine (LT4) treatment. But IVF outcomes in treated SCH women whose serum Thyroid Stimulating Hormone (TSH) concentration did and did not exceed 2.5 mIU/L before the IVF cycle has not been studied thoroughly.

**Methods:**

In this study, we performed a prospective cohort study with 270 treated subclinical hypothyroidism patients undergoing their first IVF retrieval cycle at a single cite.

**Results:**

SCH in women receiving LT4 replacement with a basal TSH level between 0.2-2.5mIU/L displayed a similar rate of clinical pregnancy (47.4% vs 38.7%, *P* = .436), miscarriage (7.4% vs 16.7%, *P* = .379) and live birth (43.9% vs 32.3%, *P* = .288) compared to women with a basal TSH level between 2.5-4.2 mIU/L.

**Conclusion:**

Strictly controlled TSH (less than 2.5 mIU/L) before IVF may have no effect on the pregnancy rate in LT4 treated SCH women.

## Background

Subclinical hypothyroidism (SCH) is a common mild thyroid disorder in women of childbearing age and may contribute to increased risk of infertility and adverse obstetric outcome of pregnancy. Observational studies examined the effect of SCH on pregnancy related outcomes, and it has been associated with multiple adverse outcomes in many, but not all, studies [1]. A recent multicenter, randomized, placebo-controlled trial found that pregnant women with treated SCH whose median TSH was 4.2 mIU/l are at the same frequencies for pregnancy loss, placental abruption, premature rupture of membranes, and neonatal death compared with untreated women [2]. Although unsound evidence exists,several professional organizations have maintained to recommend routine prenatal screening for and treatment of SCH during pregnancy [3, 4].

The alteration of serum estrogen level during pregnancy has a profound physiological impact on thyroid function. Production of thyroid hormone increases by approximately 50% during pregnancy as a normal physiological response [5]. The functional reserve of thyroid is poor in hypothyroid women substituted with levothyroxine (L-T4) and they need to increase dose of medication to maintain normal thyroid function during pregnancy. Controlled ovarian stimulation (COH) is a standard component of in vitro fertilization (IVF), which is able to induce a supra-physiological maternal serum estradiol level and may have a greater effect on thyroid function than natural conception [6, 7]. Several investigations showed that IVF had an effect on serum TSH level in L-T4 treated hypothyroid women [8]. Moreover,the majority of evidence shows that SCH has a negative effect on outcome of IVF [9]. However, whether this negative influence may be attenuated with L-T4 treatment, the benefits of strictly controlled TSH level before IVF are still unknown [3, 9]. Our aim was to investigate the rate of pregnancies of IVF in SCH women substituted with L-T4 and evaluate whether TSH less than 2.5 mIU/l is the optimal cut-off point before IVF cycle. We performed a prospective cohort study with patients undergoing their first IVF retrieval cycle at a single cite from January 2015 to March 2016 (15 months).

## Methods

### Patients

Four thousand and seven hundred twenty subfertile women who underwent either classical IVF or IVF-Intracytoplasmic Sperm Injection (ICSI) were enrolled between January 2015 and March 2016 at the first people’s hospital of Yunnan Province PR China. The level of TSH was screened and the subjects with TSH level above 4.2 mIU/L were selected, serum levels of TSH, free triiodothyronine (FT3), free thyroxine (FT4) were measured. SCH is defined as an elevated TSH concentration (TSH 4.2-20 mIU/L) with normal serum levels of FT4. When SCH was confirmed, treatment with L-T4 was started and the target TSH level is within 0.2-4.2 mIU/l. After the approval of Institutional Review Board, we selected women aged 37 years or less who underwent their first fresh IVF retrieval cycle, using their own oocytes. Two hundred and seventy SCH women substituted with LT4 were considered for further study. They were eligible if serum TSH level was within 0.2-4.2mIU/L one month proceeding the IVF cycle. Age-matched euthyroid women who underwent either classical IVF or ICSI were selected as control group in the same period. This analysis exclusively refers to the first treatment cycle.

During COH, participants were monitored closely with transvaginal ultrasound and serum hormone levels. When a lead follicle R18 mm in size was identified, intramuscular hCG was administered with oocyte retrieval 36 hours later. Three to 5 days after retrieval, embryo transfer (ET) was performed, and participants returned 14 days later for measuring serum hCG levels. Positive pregnancy test was defined as positive serum hCG, and participants were followed to determine pregnancy outcome (10 months). Samples were processed and serum stored at -20 °C for batched analysis. Exclusion criteria included those patients with ovarian hyperstimulation (E2 > 4000 pg/ml, the number of follicles over 20), poor ovarian response (less than three follicles after ovarian stimulating drugs), poor compliance. Clinical endpoints were rates of clinical pregnancy (gestational sac on ultrasound), miscarriage (loss after sonographic presence of gestational sac), and live birth (delivery confirmed via Society for Assisted Reproductive Technology and direct patient report).

### Laboratory Analysis

The levels of serum TSH, FT4, FT3 and thyroid peroxidase antibody (TPOAb) were measured with electro-chemiluminescence (ECL) immunoassays (CobasElesys 601, Roche). The TSH assay had a reference range of 0.27–4.2mIU/L with intra-assay coefficient of variation (CV) of 1.57–4.12%. For FT4, the reference range was 12-22pmol/L with intra-assay CV of 2.24%-6.33%. The FT3 reference range was 3.1-6.8pmol/L with intra-assay CV of 2.42%-5.63%. The TPOAb assay range was 5.0-1,000 IU/mL with intra-assay CV of 6.7%. Positive TPOAb status was >35 IU/mL.Positive pregnancy test was defined by serumβ-hCG more than 25mIU/ml, typically on day 12–14 after oocyte retrieval.

### Statistical analysis

Quantitative values were expressed as mean ± SD or as median and interquartile range, as appropriate. Wilcoxon Rank-Sum test (nonparametric analysis) was used for continuous data without a normal distribution; Chi-Square analysis was used for categorical data with large cell counts, the Fisher-Exact test analysis for categorical data with small cell counts, a *p*-value <0.05 was considered statistically significant.

## Results

We found in a cohort of 4720 subfertile women undergoing IVF that 5.8% of them had SCH. Two hundred and seventy SCH women were enrolled and substituted with L-T4. Ninety-four SCH women were excluded because their IVF cycle were cancelled, one hundred and seventy-six of 270 women (66.2%) completed all pregnancy study visits and included in the final analysis. Of the 4407 women (93.3%) with normal thyroid screen results, 200 were eligible and participated in the study.

Baseline characteristics of the levothyroxine treated SCH group and the euthyroid group were similar. The frequencies of adverse pregnancy outcomes did not differ significantly between the two study groups (Table 1). Eighty-two of 176 (46.6%) L-T4 treated SCH women who underwent ET had positive pregnancy tests. Seventy-eight of 176 (44.31%) became clinically pregnant. Eight pregnancies (10.3%) resulted in miscarriages (before 12 weeks of gestation). Overall rates of clinical pregnancy (44.31% vs 38.36%, *P* = .251) and miscarriage (10.3% vs 10.7%, *P* = .39) were not significantly different between L-T4 treated SCH women and euthyroid women who underwent IVF in the same period.

**Table 1:** Baseline characteristics and IVF outcomes in Treated SCH and Euthyroid Patients

	Sub-hypo women	Euthyroid women	*P*
Number of patients	*n* = 176	*n* = 200	
Age (yrs)	29.89 ± 4.07	30.45 ± 4.21	0.056
BMI(kg/m2)	22.76 ± 3.54	22.41 ± 2.89	0.099
TSH range(mIU/L)	0.32,4.21	0.57,4.21	0.420
Indication to IVF			0.439
Tubal factor infertility	50%	54.3%	
Ovulatory dysfunction	7.95%	5.9%	0.059
Endometriosis	2.2%	1.4%	
Male	15.9%	16.2%	
Mixed	21.6%	20%	
Unexplained infertility	6.5%	6.7%	
Clinical pregnancies	44.31%	38.36%	0.251
Miscarriages	10.3%	10.7%	0.39

One hundred and fourteen of 176 L-T4 treated SCH women (64.8%) had a TSH level within 0.2-2.5mIU/L before the IVF cycle. Fifty-four (47.4%) of them had positive pregnancy tests and all of them had clinical pregnancies. Fifty (43.9%) had a live birth and four (7.4%) pregnancy resulted in miscarriage. Of the remaining 62 participants with a TSH level within 2.5–4.2mIU/L before the IVF cycle, 28 (45.2%) of them had positive pregnancy tests but only 24 (38.7%) became clinically pregnant, 20 (32.3%) had a live birth, 4 (16.7%) resulting in miscarriages. The rates of clinical pregnancy (*P* = .436), miscarriages (*P* = .379) and live birth (*P* = .288) were similar between two groups. In addition, total number of oocytes retrieved (*P* = .752), good quality embryos (*P* = .752) were similar between these two groups. The baseline characteristics and cycle outcome of women whose serum TSH did and did not exceed the threshold of 2.5 mIU/L before the IVF cycle are shown in Table 2. No statistically significant differences were observed. An intragroup analysis according to thyroid autoimmunity detected also failed to document any significant result (data not shown).

**Table 2:** Baseline characteristics and IVF outcomes in women serum TSH concentration did and did not exceed 2.5 mIU/L before the IVF cycle

Characteristics	TSH ≥ 2.5mIU/L	TSH < 2.5mIU/L	*P*
	*n* = 62	*n* = 114	
Age (yrs)	30.48 ± 4.07	29.56 ± 4.37	0.336
BMI(kg/m2)	23.60 ± 4.05	22.29 ± 3.18	0.099
Previous pregnancies	48.4%	54.4%	
AFC	12.00 ± 5.00	12.00 ± 8.00	0.972
TSH(mIU/L)	2.60,4.21	0.32,1.88	0.0
FT3(pmol/L)	4.73 ± 0.76	5.10 ± 0.99	0.005
FT4(pmol/L)	17.29 ± 2.29	20.10 ± 3.99	0.001
Indication to IVF			0.439
Tubal factor infertility	45.2%	52.6%	
Ovulatory dysfunction	6.4%	8.8%	
Endometriosis	3.2%	0%	
Male	12.9%	17.5%	
Mixed	25.8%	19.3%	
Unexplained infertility	6.5%	1.8%	
Protocol of stimulation			
Long protocol	77.4%	89.5%	
Short protocol	6.5%	3.5%	
GnRH antagonists	3.2%	0%	
ART procedure			0.8
IVF	75.8%	70.2%	
ICSI	24.2%	29.8%	
Duration of stimulation(Days)	11.23 ± 1.82	11.98 ± 2.09	0.106
Total amount of recombinant FSH administration	2025 ± 825	2475.00 ± 1350.00	0.128
Serum estradiol at HCG Administration(pg/ml)	1983.50 ± 2079	2529.00 ± 2005.00	0.108
Total number of oocytes retrieved	10.00 ± 8.00	11.00 ± 8.00	0.752
Total number of good quality embryos	2.00 ± 3.00	4.00 ± 6.00	0.767
Clinical pregnancies	38.7%	47.4%	0.436
Miscarriages	16.7%	7.4%	0.379
Live birth rate	32.3%	43.9%	0.288

Data are reported as %,mean ± standard deviation or quartile,as appropriate

*AFC* antral follicle, *BMI*, body mass index, *FSH* follicle-stimulating hormone, *GnRH* gonadotropin-releasing hormone

Subgroup analyses were performed within the group of women with treated SCH. We compared women with (*n* = 102) and without (*n* = 74) thyroid autoimmunity. The median serum TSH before the IVF cycle was 1.81 (0.95–3.69) and 1.71 (0.95–2.69) mIU/L, respectively (*p* = 0.22). Baseline characteristics of the two study groups were similar with the exception of total amount of recombinant FSH, which was slightly higher in women without thyroid autoimmunity (1950.0 ± 1650.0 vs. 2400.0 ± 1950.0 IU, *p* = 0.031). The main findings of the IVF outcomes are summarized in Table 3. No statistically significant differences emerged.

**Table 3:** Baseline characteristics and IVF outcomes in women with treated subclinical hypothyroidism with or without thyroid antibodies

Characteristics	AB POS	AB NEG	*P*
	*n* = 102	*n* = 74	
Protocol of stimulation			
Long protocol	88.3%	97.3%	
Short protocol	3.9%	2.7%	
GnRH antagonists	7.8%	0%	
ART procedure			0.241
IVF	70.6%	81.1%	
ICSI	29.4%	18.9%	
Duration of stimulation(Days)	11.0 ± 10.0	12.0 ± 10.25	0.204
Total amount of recombinant FSH administration	1950.0 ± 1650.0	2400.0 ± 1950.0	0.031
Serum estradiol at HCG Administration(pg/ml)	2566.36 ± 1162.43	2269.04 ± 1239.37	0.34
Total number of oocytes retrieved	10.00 ± 6.00	11.00 ± 7.25	0.922
Total number of good quality embryos	2.00 ± 1.00	3.00 ± 1.00	0.965
Clinical pregnancies	47.1%	40.6%	0.566
Miscarriages	9.0%	18.2%	0.602
Live birth rate	43.1%	34.4%	0.291

## Discussion

Using the definition of TSH level greater than the upper limit of normal range (4-5mIU/L) with normal FT4 levels, the incidence of SCH has been reported approximately 4–8% in reproductive age population [10, 11]. Similarly, the incidence of SCH during pregnancy ranged from 2 to 2.5% [3]. Some investigators reported that SCH is more prevalent in infertile women [12, 13]. In a retrospective study, an elevated serum TSH was found in 4% of 335 infertile Finnish women with unknown history of thyroid dysfunction [8]. The incidence was highest in those with ovulatory dysfunction (6%) and unexplained infertility [5]. In this study, we enrolled 4720 subfertile women who underwent IVF for thyroid function examination. The incidence of SCH was 5.8%, using an upper cutoff TSH level of 4.2mIU/l, which is consistent with previous investigations [12, 13].

Multiple studies have reported an association of SCH with an increased risk of adverse pregnancy outcomes including placental abruption, preterm birth, fetal death, and preterm premature rupture of membranes (PPROM) [3, 14], but not all. There is fair evidence that SCH, defined as TSH outside of the normal reference range in pregnancy, is associated with miscarriage [3, 15]. Untreated SCH affected the pregnancy outcomes in women requiring IVF, such as decreased pregnancy rate, implantation rate and delivery rate. Current data have demonstrated some benefits of L-T4 treatment in reducing these events [9]. In 2007, investigators performed a randomized trial on a population of 70 infertile patients with SCH who had undergone IVF. Patients were randomly assigned to either the L-T4 treatment group (50–100 mc g L-T4 starting at one month before the assisted reproduction technology procedure) or the placebo group. They found that compared with untreated group, the miscarriage rate was significantly lower (9% vs 13%, respectively) and the clinical pregnancy rate and delivery rate were significantly higher (35% and 10% vs 26% and 3%, respectively) in L-T4 treatment group[16]. Another study randomized 64 women with SCH to the L-T4 treatment group (50 mcg L-T4 starting at day 1 of ovarian stimulation) or the control group [17]. They observed a significant increase in the number of grade I or II embryos (*P* = .007) and in the implantation rate (26.9% vs 14.9%, *P* = .044). The miscarriage rate was significantly lower in the L-T4 treatment group (0 vs 33.3%; *P* = .021) and as a result, the live-birth rate was significantly higher in the L-T4 treatment group (53.1% vs 25%, *P* = .039). In our study, 176 SCH women were substituted with L-T4 and their TSH levels were maintained within 0.2-4.2 mIU/L one month before the IVF cycle. We found that the rate of positive pregnancy test, clinical pregnancies and miscarriage were similar between treated SCH women and euthyroid women who underwent IVF, which is consistent with previous investigations. The investigation found that IVF outcome was not significantly hampered in women with adequately treated hypothyroidism [18].

There is powerful evidence that L-T4 treatment in women with SCH is associated with improvement in pregnancy and miscarriage rates [1], but, the benefit of thyroid hormone use on pregnancy loss was maily observed among women with basal TSH concentrations of 4.1–10.0 mIU/L, not those with concentrations of 2.5-4.0 mIU/L. Furthermore, the benefits of strict TSH control remains to be determined [4, 19–21]. Few studies have examined if the risks of adverse obstetric outcomes are increased with a TSH level higher than 2.5mIU/L but less than the upper range of normal at the time of conception in IVF. Although unsound evidence exists recent Clinical Practice Guideline by The Endocrine Society suggests TSH levels in subclinical women should be less than 2.5 mIU/L before pregnancy, particularly in women requiring IVF [4]. One study measured 1231 women pursuing assisted reproductive technologies (ART). In this study, investigators found no evidence of an elevated pregnancy loss rate in women with preconception elevated TSH (2.5–4.0 mIU/L) [22]. The observation is in agreement with a recent study by Reh et al. who found no difference in clinical pregnancy, delivery, or miscarriage in patients with mildly elevated basal TSH (>2.5 and <4.0 mIU/L) compared to patients with a normal TSH (>0.4 to <2.5 mIU/L) [20]. Other studies suggest that there is a trend toward increasing risk of miscarriage with increasing TSH level, although this trend did not reach statistical significance [21]. These controversal results raise questions about the current guideline recommended threshold of 2.5 mIU/L for treating SCH when thyroid antibody was positive. In our study, no differences in the rates of positive pregnancy test, clinical pregnancies, miscarriage or live birth were observed in treated SCH women with different TSH level. There was no significant difference between the two groups with the number of oocytes retrieved and in the implantation rate. In addition, we also use TSH values greater than 2.5 mIU/L on HCG admistration day as a cut-off point. There were no differences in the rates of positive pregnancy test, clinical pregnancies, miscarriage or live birth in different groups (data not shown).

However, due to the small sample size, the statistical power of this study was limited. In addition, since we didn’t adjust the levothyroxine dose during COH, we cannot determine whether there is an association between TSH alteration during ART and IVF outcome. We didn’t measure urinary iodine in this study that was also a flaw. Finally, according to ATA guideline, we arbitrarily chose 2.5 mIU/L as a strictly controlled TSH threshold. It would have been more appropriate to refer to a local threshold since reference range may vary among different ethnics and is not always correct to use the same threshold for all ethnics.

## Conclusion

In conclusion, our results suggest that L-T4 treatment in women with SCH requiring IVF had similar rates of pregnancy and adverse pregnancy outcomes compared to euthyroid women. Strictly controlled TSH (less than 2.5 mIU/L) before IVF may have no effect on the pregnancy rate and maternal adverse pregnancy results. Whether the risk of other adverse outcomes could be attenuated calls for further studies to evaluate the effectiveness of thyroid hormone treatment in this population.

## Abbreviations

ART: Assisted Reproductive Technology
COH: Controlled ovarian stimulation
ET: Embryo Transfer
FT3: Free Triiodothyronine
FT4: Free Thyroxine
ICSI: Intracytoplasmic Sperm Injection
IVF: in vitro fertilization
L-T4: Levothyroxine
SCH: Subclinical hypothyroidism
